# Acute occlusion of the LMCA without significant dynamic changes in the ECG: A case report

**DOI:** 10.1097/MD.0000000000043809

**Published:** 2025-08-08

**Authors:** Huiqi Zhai, Rong Li, Xinjun Zhao, Qingmin Chu

**Affiliations:** a Department of Cardiovascular Disease, The First Clinical Medical College of Guangzhou University of Chinese Medicine, Guangzhou, China; b Department of Cardiovascular Disease, The First Affiliated Hospital of Guangzhou University of Chinese Medicine, Guangzhou, China.

**Keywords:** case report, electrocardiogram, left main coronary artery occlusion, percutaneous coronary intervention

## Abstract

**Rationale:**

Acute myocardial infarction caused by left main coronary artery (LMCA) occlusion is a critical condition with high mortality. However, when it presents with atypical symptoms and lacks dynamic electrocardiogram (ECG) changes, it poses a diagnostic challenge leading to delayed treatment and poor outcomes. This case report aims to highlight the importance of early recognition and intervention in such “double-negative” presentations to improve prognosis and reduce mortality.

**Patient concerns:**

A 49-year-old male with no cardiac history presented to the emergency department with abdominal distension for 4 hours after ingesting a large amount of alcohol.

**Diagnoses:**

Acute complete occlusion of the LMCA.

**Intervention:**

Percutaneous coronary intervention.

**Outcomes:**

One Firebird 3.0 × 33 mm (MicroPort company, Shanghai China) stent was successfully implanted in the LMCA, and the stenosis disappeared and blood flow was smooth after the operation.

**Lessons:**

A typical AMI without dynamic ECG changes requires aggressive diagnostic workup, including early angiography, to prevent fatal outcomes.

## 1. Introduction

Acute myocardial infarction (AMI), recognized as the leading global cause of cardiovascular mortality, represents a critical clinical syndrome characterized by myocardial necrosis secondary to acute coronary occlusion, ultimately resulting in impaired cardiac systolic function.^[1]^ Among AMI subtypes, left main coronary artery (LMCA) myocardial infarction is acknowledged as the most lethal variant due to its involvement of >75% of the left ventricular myocardial blood supply. The classic presentation typically features retrosternal crushing pain accompanied by dynamic electrocardiographic (ECG) evolution (ST-segment elevation/*Q*-wave formation) and elevated cardiac biomarkers. Notably, about 20.0% of AMI cases manifest with atypical symptoms (e.g., epigastric distress, pharyngeal constriction, or mandibular referred pain), among which only a few exhibit concurrent absence of dynamic ECG changes – a “double-negative” presentation that substantially increases diagnostic uncertainty.^[2]^ In this paper, we report a diagnostically challenging LMCA-AMI case exhibiting both atypical symptomatology and static ECG findings, subsequently confirmed by coronary angiography and managed with emergency revascularization.

## 2. Case presentation

**Figure 1.**
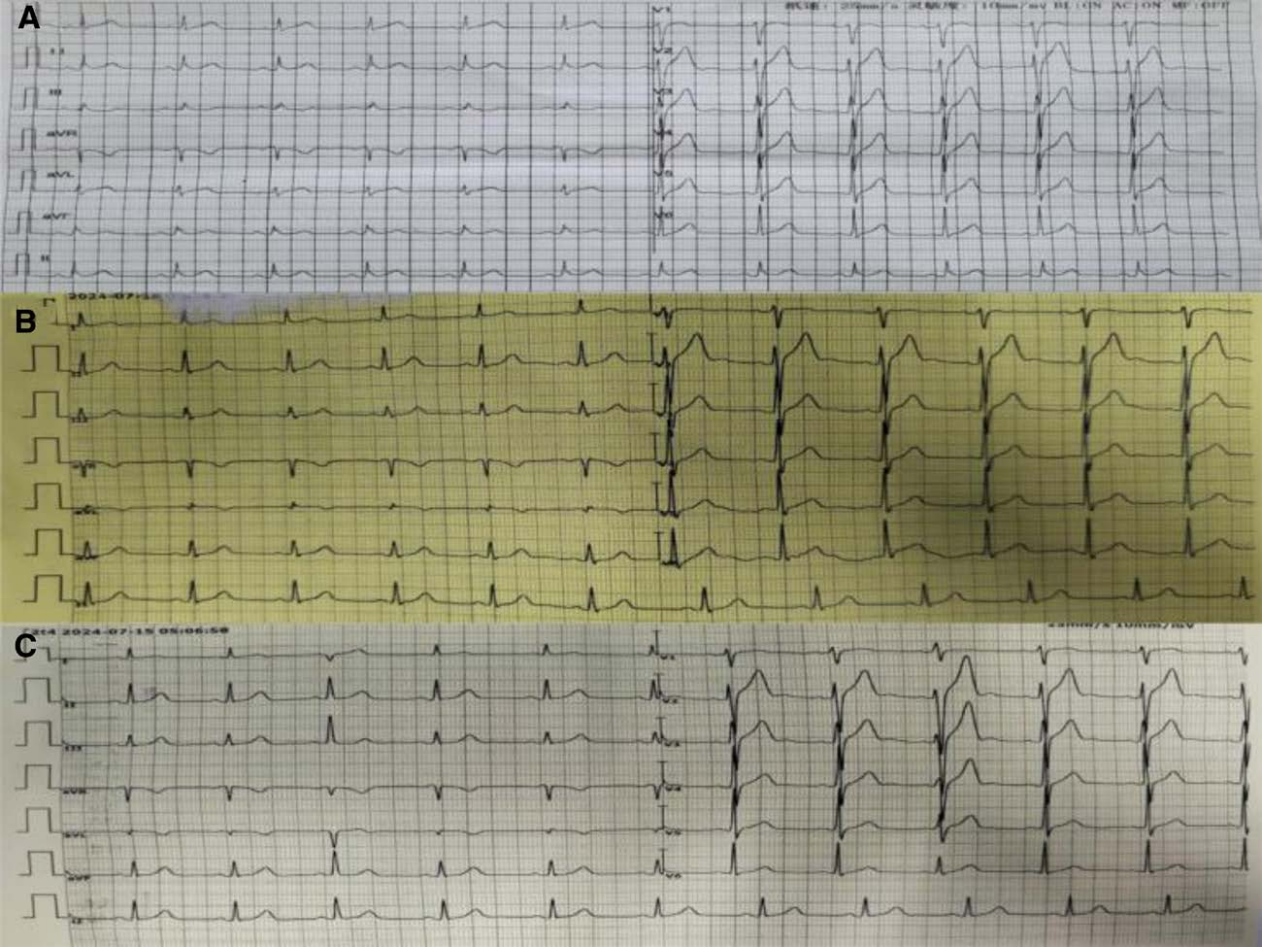
Patient’s electrocardiograms. (A) Patient’s usual ECG. (B) Patient’s first ECG in the emergency room. (C) Patient’s 2nd ECG in the emergency room. ECG = electrocardiogram.

A 49-year-old male with no cardiac history and no other medical history. He presented to the emergency department complaining of abdominal distension for 4 hours. The patient complained that his malaise arose after ingesting a large amount of alcohol. He took magnesium aluminum carbonate on his own, which provided no relief. In the emergency department, his blood pressure was 104/78 mm Hg, heart rate was 67 beats/min, and oxygen saturation was 98%. The patient underwent an electrocardiogram (ECG) and troponin test in the emergency department. The 2 electrocardiograms did not show significant changes compared to the patient’s previous physical examination electrocardiogram (Fig. 1). But his ultrasensitive troponin I test came back at 1.560 ng/mL (Ref 0–0.04 ng/mL).

## 3. Outcomes

**Figure 2.**
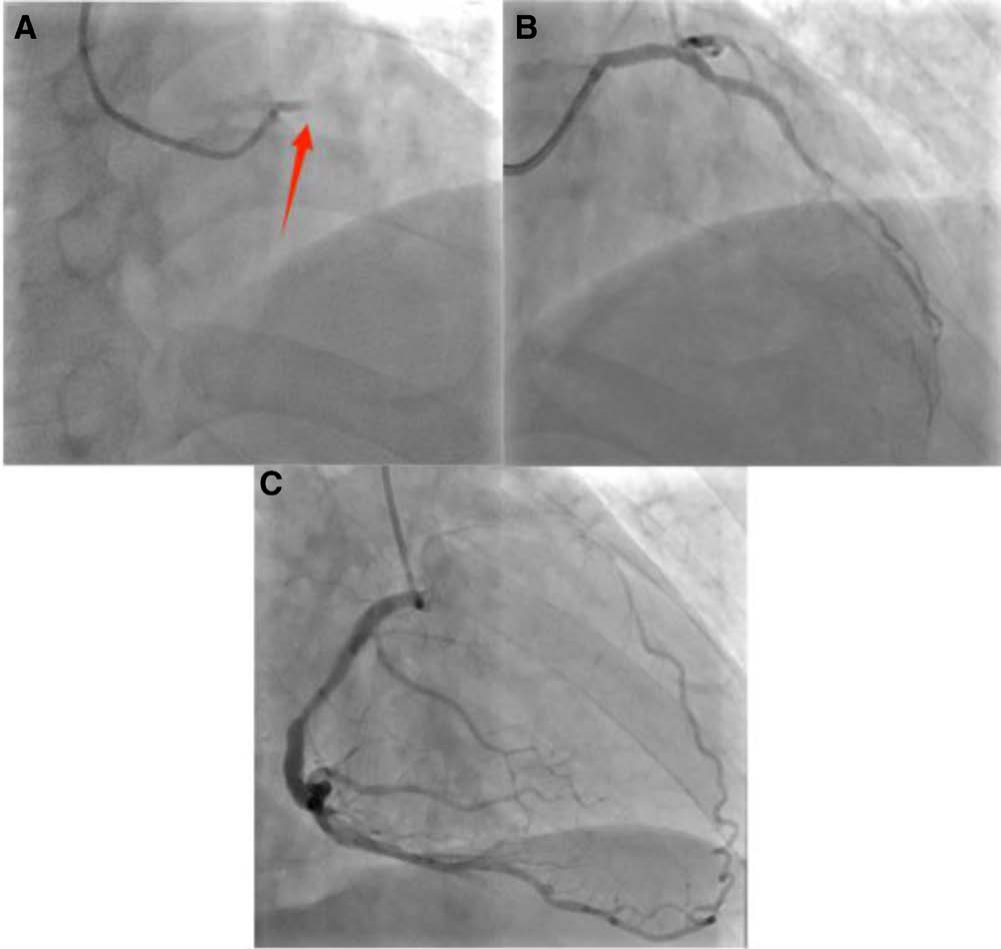
Coronary angiogram of the patient. (A) Middle LMCA occlusion. (B) The state of an occluded coronary artery (LMCA) after it has been opened. (C) Patient’s thick RCA. LMCA = left main coronary artery, RCA = right coronary artery.

Emergency percutaneous coronary angiography revealed acute total occlusion of the proximal segment of the LMCA (Fig. 2A). The patient’s right coronary artery was dominant, with collateral branches supplying the ischemic territory distal to the LMCA occlusion (Fig. 2C). Percutaneous coronary intervention was performed immediately: a Firebird 3.0 × 33 mm stent (MicroPort, Shanghai, China) was deployed in the LMCA, achieving TIMI grade 3 flow and complete resolution of stenosis (Fig. 2B).

Post-procedure, ultrasensitive troponin I peaked at 8.42 ng/mL within 12 hours but normalized to 0.03 ng/mL by day 5. Transthoracic echocardiography on day 3 showed mild anterior hypokinesis (LVEF 53%). The patient was discharged on day 7 on dual antiplatelet therapy (aspirin 100 mg/day, ticagrelor 90 mg twice daily), atorvastatin 20 mg/day, pantoprazole sodium enteric-coated tablets 40 mg/day, metoprolol succinate 47.5 mg/day and so on. At 3-month follow-up, he reported no cardiac symptoms, and repeat echocardiography demonstrated restored LVEF (65%) without residual wall motion abnormalities.

## 4. Discussion

AMI is an important cause of death worldwide.^[3]^ Myocardial infarction with LMCA occlusion is the more aggressive of all infarctions and is often accompanied by significant changes in the electrocardiogram. For example, the “6 + 2” phenomenon, STaVR ↑ and STaVR ↑>STV1 ↑, and new-onset right bundle branch block and so on.^[4]^ LMCA thrombosis with AMI is a rare condition with very high mortality.^[5]^ In patients undergoing coronary angiography, the incidence of complete occlusion of the LMCA is roughly 0.04% to 0.42%,^[6]^ and its actual incidence is usually higher than the clinical statistics because of its high mortality rate and the fact that many of the patients have already suffered a sudden death before hospitalization. Thus, LMCA occlusion is a very rare and critical type of coronary artery lesion that deserves high attention.

AMI typically presents with hallmark symptoms such as chest pain/pressure, a sense of impending doom, and characteristic ECG changes accompanied by mild or significant elevations in cardiac biomarkers. Undiagnosed AMI in such atypical presentations carries substantial prognostic implications, as delayed recognition and subsequent undertreatment significantly contribute to poor clinical outcomes. This case highlights that AMI may occur even in the absence of classic symptoms or definitive ECG evolutionary patterns. The absence of dynamic ECG changes despite complete LMCA occlusion was likely attributable to robust collateral circulation from the patient’s dominant right coronary artery, which perfused the ischemic myocardium subtended by the occluded LMCA (Fig. 2). This phenomenon underscores that well-developed collaterals could mask classical ischemic ECG patterns, leading to under-recognition of critical coronary lesions.^[7]^ Interestingly, acute alcohol intake may influence the generation of new collateral circulation. Koerselman et al demonstrated a J-shaped association between alcohol intake and coronary collateral presence, suggesting low-to-moderate alcohol exposure might enhance collateralization.^[8]^ While speculative, this mechanism could partly explain the preserved myocardial perfusion and static ECG in our patient following excessive alcohol ingestion.

To mitigate diagnostic challenges, clinicians should implement serial monitoring of ECG tracings and cardiac biomarkers (e.g., troponin, CK-MB) in patients with suspicious clinical manifestations, regardless of initial biomarker levels or static ECG findings. When clinical suspicion persists despite inconclusive noninvasive testing, coronary angiography should be strongly considered to confirm diagnosis, particularly in high-risk populations. Enhanced vigilance for atypical AMI presentations combined with proactive diagnostic strategies could improve detection rates and optimize time-sensitive therapeutic interventions. For example, Had echocardiography preceded troponin testing, it might have revealed regional wall motion abnormalities (e.g., anterior/apical hypokinesis), potentially accelerating invasive evaluation. Conversely, had angiography been negative, alternative etiologies for troponin elevation (e.g., demand ischemia, myocarditis) would have been pursued, particularly given the atypical presentation. Ultimately, in high-suspicion scenarios like LMCA occlusion, early angiography remains pivotal despite ambiguous noninvasive findings.

## 5. Limitations

This case report a single case, limiting generalizability. The relationship between acute alcohol excess and AMI pathogenesis (e.g., platelet activation, endothelial dysfunction) was not explored. While we hypothesize alcohol-induced collateralization masked ECG changes, this remains speculative and warrants mechanistic studies. Additionally, advanced imaging (e.g., cardiac MRI) was unavailable to quantify microvascular injury. Future multi-center studies with larger cohorts are needed to validate these observations.

## Author contributions

**Conceptualization:** Huiqi Zhai, Rong Li, Qingmin Chu.

**Data curation:** Huiqi Zhai, Rong Li, Xinjun Zhao, Qingmin Chu.

**Formal analysis:** Huiqi Zhai, Rong Li, Xinjun Zhao, Qingmin Chu.

**Funding acquisition:** Huiqi Zhai, Qingmin Chu.

**Investigation:** Huiqi Zhai, Rong Li, Xinjun Zhao.

**Methodology:** Huiqi Zhai.

**Project administration:** Huiqi Zhai, Xinjun Zhao, Qingmin Chu.

**Resources:** Qingmin Chu.

**Software:** Huiqi Zhai.

**Supervision:** Huiqi Zhai, Rong Li, Xinjun Zhao.

**Validation:** Huiqi Zhai, Qingmin Chu.

**Visualization:** Huiqi Zhai, Qingmin Chu.

**Writing – original draft:** Huiqi Zhai.

**Writing – review & editing:** Huiqi Zhai, Rong Li, Xinjun Zhao, Qingmin Chu.
